# Older Primary Care Patients' Attitudes and Willingness to Screen for Dementia

**DOI:** 10.1155/2015/423265

**Published:** 2015-04-20

**Authors:** Nicole R. Fowler, Anthony J. Perkins, Hilary A. Turchan, Amie Frame, Patrick Monahan, Sujuan Gao, Malaz A. Boustani

**Affiliations:** ^1^Indiana University Center for Aging Research, Indianapolis, IN 46202, USA; ^2^Regenstrief Institute, Inc., Indianapolis, IN 46202, USA; ^3^Indiana University School of Medicine, Indianapolis, IN 46202, USA; ^4^Indiana University Department of Biostatistics, Indianapolis, IN 46202, USA

## Abstract

*Objective*. To understand older primary care patients' perceptions of the risks and benefits of dementia screening and to measure the association between attitudes and screening behaviors. *Methods*. Eligible patients completed the Perceptions Regarding Investigational Screening for Memory in Primary Care (PRISM-PC) questionnaire and then were asked to undergo dementia screening by a telephone screening instrument. *Results*. Higher scores on the PRISM-PC questionnaire items that measure attitudes about benefits of screening were associated with decreased odds of refusing screening. Participants who refused screening had significantly lower PRISM-PC questionnaire scores on the items that measure perceived benefits compared to those who agreed to screening. Participants who refused screening were less likely to agree on screening for other conditions, such as depression and cancer. Participants who know someone with Alzheimer's disease (AD) were less likely to refuse screening. *Discussion*. Patients' attitudes about the benefits of dementia screening are associated with their acceptance of dementia screening.

## 1. Introduction

It is estimated that by 2050 there will be 11 to 19 million people in the United States of America (USA) living with dementia [1]. The incidence of dementia is also growing globally with a new patient being diagnosed approximately every 7 seconds [2]. Although there is currently no cure or proven prevention strategies for dementia, pharmacological and nonpharmacological interventions are available that may impact the symptoms of dementia [3, 4].

Despite rising incidence rates, many patients with dementia go unrecognized and never receive a cognitive evaluation or diagnosis. Estimates of undiagnosed dementia among older adults in the USA range from 45% to 80% [3, 5–7]. In 2013 the United States Preventive Services Task Force (USPSTF) concluded that the evidence to routinely screen for dementia in primary care is insufficient due to a lack of studies evaluating the risks, benefits, and patient perspectives of the value of dementia screening [8]. Understanding patients' attitudes about the risks and benefits of early identification of dementia is vital to assess the value of population-based dementia screening. In addition, this information allows for a better understanding of potential barriers and facilitators in the implementation of dementia screening programs in primary care settings [9].

The Perceptions Regarding Investigational Screening for Memory in Primary Care (PRISM-PC) questionnaire was developed by researchers at the Indiana University Center for Aging Research to identify the attitudes of older adults regarding screening for dementia in primary care. The purpose of this study is to understand primary care patients' perceptions of the risks and benefits of screening and early identification of dementia and to measure the association between patients' attitudes and behaviors of screening [10]. Based on our previous work, we hypothesized that many patients would report perceived benefits of early identification of dementia and that patient's acceptance of screening would be associated with the belief that early identification improves the patient-centered outcomes of the disease. Conversely, we hypothesized that refusal to be screened would be associated with the fear that early detection would result in being stigmatized or the loss of independence.

## 2. Methods

### 2.1. Study Population

Eligible patients were 65 years and older, had no diagnosis of dementia, had seen their primary care physician within the previous 12 months, and received their primary care at either St. Vincent Health or Community Health Network, both of which are located in Indianapolis, IN. Patients were excluded if they did not speak English, had hearing loss that precluded them from communicating via telephone, and had severe mental illness indicated in their medical record. This study was approved by the Institutional Review Boards of St. Vincent Hospital and the Community Health Network.

The research team obtained a list of eligible patients from the St. Vincent Health and Community Heath primary care office staff and appointment scheduling records. Eligible patients at the two sites were approached by telephone and offered the opportunity to participate in the study by research assistants from the Indiana University Center for Aging Research. All recruitment procedures complied with the Health Insurance Portability and Accountability Act and Institutional Review Board regulations. Informed consent was obtained from all patients who agreed to participate in the study.

### 2.2. Study Procedures and Instruments

The study uses The Perceptions Regarding Investigational Screening for Memory in Primary Care (PRISM-PC) questionnaire to measure patient's attitudes and perceived harms and benefits of screening for dementia. The PRISM-PC questionnaire includes 50 items with 12 items of capturing self-reported sociodemographic data and information regarding a participant's experience with or exposure to Alzheimer's disease and 38 items measuring the participant's perceptions of the acceptability, harms, and benefits of dementia screening. The PRISM-PC questionnaire uses the term Alzheimer's disease as a substitute for dementia since previous studies have shown that patients are more familiar with the term “Alzheimer's disease” than “dementia.” Each item of the questionnaire is scored on a 1- to 5-point Likert scale with possible responses of strongly agree, agree, do not know, disagree, and strongly disagree for the following domains: benefits of dementia screening (8 items), stigma of dementia screening (10 items), negative effect of dementia screening on independence (6 items), suffering related to dementia screening (4 items), perceived acceptance of different types of dementia screening (6 items), perceived acceptance of screening for other conditions—colon cancer and depression (2 items), and the belief that a treatment for Alzheimer's disease is not currently available (2 items) [10].

IU-PBRN research assistants approached eligible individuals via telephone and explained the study. After obtaining consent, research assistant administered the PRISM-PC questionnaire and asked participants to undergo dementia screening. If participants agreed to screening, it was conducted during the same phone interview using the Telephone Instrument for Cognitive Screening (TICS). Participants who scored ≤ 30 screened positive for dementia and were referred to a specialist at either St. Vincent Health or Community Health Network for an evaluation and diagnostic assessment.

### 2.3. Statistical Analyses

Prior to conducting the analyses, responses on the PRISM-PC questionnaire were reverse-coded so that a higher score indicated stronger agreement on the items. To facilitate interpretation of the domain scores, we used a similar approach that is used to analyze scale scores on the SF-36 [11]. We converted all domains to the same metric by taking the sum of the reverse-coded responses and then transforming the sum to a 0 to 100 scale by subtracting the minimum possible score and dividing by the possible range. For a given domain, this meant that 0 represented strongly disagree on all items, 100 represented strongly agree on all items, and 50 represented neutral scores on all items.

For comparisons of groups of participants, we used *t* tests if the variables were continuous and Fisher's exact tests if the variables were categorical. To model the association of the PRISM-PC questionnaire domains with the dependent variable (acceptance versus refusal of telephone screening with the TICS), we used logistic regression and adjusted for covariates found to be significant or marginally significant (*P* < 0.10) in bivariate analyses. We reported the results in terms of odds ratios (ORs) and confidence intervals (CIs).

To determine if the scales derived from the phone administration of the PRISM-PC questionnaire showed the same internal consistency from prior studies when the PRISM-PC questionnaire was administered in person, we calculated Cronbach's alpha for each scale and compared it to previously published PRSIM-PC questionnaire findings [12]. For all statistical analyses, we used SAS statistical software version 9.3 (SAS Institute Inc., Cary, North Carolina).

## 3. Results

Sociodemographic characteristics and participants' experience with Alzheimer's disease are presented by study site in Table 1. There were no significant differences between the two study sites regarding prior experience with Alzheimer's disease. There were several significant differences between sites in sociodemographic characteristics. Participants from St. Vincent Health were significantly more likely to be married (*P* < 0.05), have higher levels of education (*P* < 0.01), and more likely to be African American (*P* < 0.001). Community Health Network participants tended to be older (*P* = 0.05) and more likely to live alone (*P* = 0.06).

Cronbach's alphas for the PRISM-PC questionnaire domain scales at the St. Vincent Health site were 0.85 for benefits of dementia screening, 0.74 for stigma, 0.72 for loss of independence, and 0.61 for suffering. Cronbach's alphas for the Community Health Network sample were 0.81 for benefits, 0.78 for stigma, 0.78 for loss of independence, and 0.47 for suffering. These Cronbach alphas are comparable to or greater than the Cronbach alphas calculated from similar samples and in previously published PRISM-PC questionnaire studies when patients completed the PRISM-PC questionnaire in person and were asked to undergo dementia screening in person [10, 12].

The associations of demographic characteristics and prior AD experience with screening refusal are presented by site in Table 2. Overall there were no significant differences between dementia screening refusal at the two sites, 37.7% at St. Vincent and 36.1% at Community (*P* = 0.746). The only demographic variable significantly associated with screening refusal at the St. Vincent Health site was education. At St. Vincent Health only, participants with less than a high school education were more likely to refuse screening than the participants with at least a high school education. At both sites, participants without a relative or friend with AD were significantly more likely to refuse the screening (St. Vincent Health 45.8% versus 28.2%; *P* = 0.003 and Community Health Network 42.1% versus 26.1%; *P* = 0.074).

The association between screening refusal and the four domains on the PRISM-PC questionnaire and on individual questionnaire items are presented in Table 3. For both sites, participants who refused screening had significantly lower scores on the benefits domain than participants who agreed to screening. In addition, participants who refused screening at both sites were less likely to agree to question on the PRISM-PC questionnaire regarding annual screening for depression. At the Community Health Network site, participants who refused screening had significantly lower scores on the suffering domain, meaning they were more likely to agree on the items related to suffering as a result of screening for dementia.

Results from the logistic regressions are presented in Table 4. After adjusting for age and education, higher scores on the items in the benefits domain were significantly associated with decreased odds of refusing dementia screening at both sites. Additionally, at both sites, respondents who endorsed having a friend or relative with AD were less likely to refuse screening (St. Vincent Health AOR = 0.49; *P* = 0.009 and Community Health Network AOR = 0.39; *P* = 0.052).

## 4. Discussion

In our study of primary care patients at two different Indianapolis health care centers, we found that, despite different sociodemographic characteristics, predictors of who accepts and who declines dementia screening are similar. In this study, where participants completed the PRISM-PC questionnaire and dementia screening by phone, perceptions about the benefits of early identification remained a significant predictor of screening acceptance as did having a friend or relative with dementia. These results are consistent with previous studies that have administered the PRISM-PC questionnaire in person and administered dementia screening in person.

Findings form this study corroborate previous results that have shown that people's perceptions about the benefits of dementia screening are associated with their willingness to be screened. For example, the participants who had stronger agreement on the statements regarding the benefits of knowing about dementia earlier (e.g., ability to plan for the future) were more likely to accept screening. Of the various items of sociodemographic data that were gathered for our study, only level of education at the St. Vincent's Health System site was highly predictive of agreeing to be screened. The participants from the St. Vincent's Health System with less than a high school education were more likely to refuse screening than those with more than a high school education.

This study is the first to measure attitudes about dementia screening with the PRISM-PC questionnaire and offer screening by phone. The internal consistency reliability of the domain scales as measured by Cohen's Kappa was similar to those obtained from face-to-face administration of the PRISM-PC questionnaire and to those obtained in prior studies. In our sample, more than half (63.7%) of older primary care patients agreed to be screened for dementia by phone following completion of the PRISM-PC questionnaire. Despite the majority of participants agreeing, this rate is significantly less than our previous work that has found rates of people willing to be screened as high as 89.7% when we approached in person and screened in person [10].

A limitation of this study is that we do not know if participants had been screened for dementia as part of routine care, prior to being recruited for this study. However, none had a dementia diagnosis and less than 2% reported being told by their physician that they had memory problems.

In summary, the PRISM-PC questionnaire instrument is a valid tool to assess older primary care patients' perceptions about dementia screening, even when administered by phone. Across our work, in this study and others, belief in the benefits of recognizing dementia early is an important predictor of patient behaviors regarding screening and could be used in interventions designed to increase the uptake of dementia screening.

## Figures and Tables

**Table 1:** Sociodemographic characteristics and experience with Alzheimer's disease of study participants from St. Vincent Health and Community Health Network.

	St. Vincent Health (*n* = 278) %	Community Health Network (*n* = 122) %	*P* value
Age, years			0.051
65–69	32.3	20.5	
70–74	20.6	22.1	
75–79	20.6	19.7	
≥80	26.5	37.7	
Gender			0.172
Female	66.9	73.8	
Race			<0.001
White	78.1	96.7	
African American	20.9	2.5	
Other	1.1	0.8	
Education, years			0.003
0–11	4.3	12.3	
≥12	95.7	87.7	
Housing status			0.063
Living alone	33.7	43.4	
Living with someone	66.3	56.6	
Marital status			0.030
Married	55.8	45.1	
Widowed	28.3	43.4	
Divorced	11.6	8.2	
Never married	4.3	3.3	
Income			0.124
<$10,000	4.1	5.1	
$10,000–$19,999	17.3	20.4	
$20,000–$39,999	28.4	38.8	
≥$40,000	50.2	35.7	
Do you have a relative or friend with Alzheimer's disease?			0.189
Yes	44.8	37.7	
Do you believe that you are at higher risk of Alzheimer's disease than others of your same age?			0.243
Yes	13.2	9.1	
Do you think you have more memory problems than others of your same age?			0.348
Yes	7.6	5.0	
Has a doctor told you that you have memory problems?			0.911
Yes	1.8	1.6	
Are you taking medication to help with memory?			0.733
Yes	2.2	1.6	

**Table 2:** Bivariate comparison of the sociodemographic characteristics and experience with Alzheimer's disease of study participants who accepted and refused screening for dementia^a^ at St. Vincent Health and Community Health Network.

	St. Vincent Health (*n* = 278)			Community Health Network (*n* = 122)		
	Number (%) accepted	Number (%) refused	*P* value	Number (%) accepted	Number (%) refused	*P* value
Overall	173 (62.2)	105 (37.8)		78 (63.9)	44 (36.1)	
Age, years			0.780			0.071
65–69	55 (61.8)	34 (38.2)		19 (76.0)	6 (24.0)	
70–74	39 (68.4)	18 (31.6)		18 (66.7)	9 (33.3)	
75–79	34 (59.6)	23 (40.4)		16 (66.7)	8 (33.3)	
≥80	45 (61.6)	28 (38.4)		25 (54.4)	21 (45.6)	
Gender			0.844			0.292
Female	115 (61.8)	71 (38.2)		60 (66.7)	30 (33.3)	
Male	58 (63.0)	34 (37.0)		18 (56.2)	14 (43.8)	
Race			0.541			0.748
White	137 (63.1)	80 (36.9)		75 (63.6)	43 (36.4)	
African American	35 (60.3)	23 (39.7)		2 (66.3)	1 (33.3)	
Other	1 (33.3)	2 (66.7)		1 (100.0)	0 (0.0)	
Education, years			0.007			0.814
0–11	3 (25.0)	9 (75.0)		10 (66.7)	5 (33.3)	
≥12	170 (63.9)	96 (36.1)		68 (63.6)	39 (36.4)	
Housing status			0.733			0.063
Living alone	57 (61.3)	36 (38.7)		29 (54.7)	24 (45.3)	
Living with someone	116 (63.4)	67 (36.6)		49 (71.0)	20 (29.0)	
Marital status			0.550			0.865
Married	98 (63.6)	56 (36.4)		37 (67.3)	18 (32.7)	
Widowed	48 (61.5)	30 (38.5)		33 (62.3)	20 (37.7)	
Divorced	17 (53.1)	15 (46.9)		6 (60.0)	4 (40.0)	
Never married	9 (75.0)	3 (25.0)		2 (50.0)	2 (50.0)	
Income (*N* = 465)			0.122			0.205
<$10,000	5 (62.5)	3 (37.5)		4 (80.0)	1 (20.0)	
$10,000–$19,999	18 (52.9)	16 (47.1)		15 (75.0)	5 (25.0)	
$20,000–$39,999	41 (73.2)	15 (26.8)		20 (52.6)	18 (47.4)	
≥$40,000	73 (73.7)	26 (26.3)		25 (71.4)	10 (28.6)	
Do you have a relative or friend with Alzheimer's disease?			0.003			0.074
No	83 (54.2)	70 (45.8)		44 (57.9)	32 (42.1)	
Yes	89 (71.8)	35 (28.2)		34 (73.9)	12 (26.1)	
Do you believe that you are at higher risk of Alzheimer's disease than others of your same age?			0.106			0.511
No	144 (61.0)	92 (39.0)		69 (62.7)	41 (37.3)	
Yes	27 (75.0)	9 (25.0)		8 (72.7)	3 (27.3)	
Do you think you have more memory problems than others of your same age?			0.653			0.896
No	158 (61.7)	98 (38.3)		73 (64.0)	41 (36.0)	
Yes	14 (66.7)	7 (33.3)		4 (66.7)	2 (33.3)	
Has a doctor told you that you have memory problems?			0.917			0.679
No	170 (62.3)	103 (37.7)		77 (64.2)	43 (35.8)	
Yes	3 (60.0)	2 (40.0)		1 (50.0)	1 (50.0)	
Are you taking medication to help with memory?			0.821			0.679
No	169 (62.1)	103 (37.9)		77 (64.2)	43 (35.8)	
Yes	4 (66.7)	2 (33.3)		1 (50.0)	1 (50.0)	

^a^Because our early work indicated that patients more readily understood the term “Alzheimer's disease” than the term “dementia,” in this study we used Alzheimer's disease as a proxy for dementia.

**Table 3:** Bivariate comparison of the mean PRISM-PC questionnaire scores of study participants who accepted and refused screening for dementia at St. Vincent (St. V.) Health and Community Health Network (Community).

Domains and individual items^a^	St. V. Health			Community		
	Mean score (SD)		*P* Value	Mean score (SD)		*P* Value
	Accepted screening (*n* = 173)	Refused screening (*n* = 105)		Accepted screening (*n* = 78)	Refused screening (*n* = 44)	
Domain: benefits of dementia screening	73.8 (10.1)	67.7 (13.3)	<0.001	72.1 (9.5)	65.6 (12.8)	0.002
Domain: stigma of dementia screening	32.0 (11.4)	33.3 (9.2)	0.323	33.7 (11.5)	34.9 (9.3)	0.545
Domain: negative impact of dementia screening on independence	50.6 (12.6)	53.3 (13.8)	0.086	50.8 (11.1)	49.1 (11.8)	0.444
Domain: suffering related to dementia screening	59.0 (13.5)	58.6 (14.9)	0.828	59.1 (11.9)	53.0 (12.2)	0.009
Item in no domain: agreement with screening for colon cancer	3.3 (1.0)	3.2 (1.0)	0.242	3.2 (1.1)	2.8 (1.0)	0.083
Item in no domain: agreement with screening for depression	3.2 (1.0)	2.8 (1.0)	0.007	3.3 (1.0)	2.8 (1.0)	0.006
Item in no domain: belief that a treatment for Alzheimer's disease is not currently available	2.6 (0.9)	2.7 (0.8)	0.281	2.5 (0.9)	2.7 (0.7)	0.217

PRISM-PC: Perceptions Regarding Investigational Screening for Memory in Primary Care.

^a^For each domain, the table includes the individual item that was most relevant to our study objectives. The table also includes the 3 individual items that are not covered under any domain.

**Table 4:** Logistic regression analysis of the odds of refusing to undergo screening for dementia at St. Vincent Health and Community Health Network.

Variable	St. Vincent Health		Community Health Network	
	Odds ratio (95% confidence interval)	*P* value	Odds ratio^a^ (95% confidence interval)	*P* value
PRISM-PC questionnaire items				
High domain score: perception that dementia screening is beneficial	0.80 (0.70, 0.91)	0.001	0.79 (0.63, 0.98)	0.029
High domain score: suffering related to dementia screening	1.07 (0.97, 1.18)	0.200	0.89 (0.73, 1.08)	0.248
Perception that depression screening is beneficial	0.80 (0.62, 1.04)	0.100	0.63 (0.40, 0.99)	0.049
Have a relative or friend with AD	0.51 (0.30, 0.87)	0.014	0.43 (0.16, 1.12)	0.082
Lives alone	1.14 (0.64, 2.02)	0.662	2.18 (0.87, 5.44)	0.096
Education				
(>high school+ versus 0–11 years)	0.19 (0.05, 0.82)	0.025	1.38 (0.36, 5.25)	0.640
Age, years		0.648		0.573
65–69 (reference group)	1.00 [reference]		1.00 [reference]	
70–74	0.69 (0.32, 1.49)		1.71 (0.42, 6.93)	
75–79	1.17 (0.56, 2.45)		1.02 (0.24, 4.74)	
≥80	0.90 (0.44, 1.82)		2.01 (0.56, 7.25)	

PRISM-PC, Perceptions Regarding Investigational Screening for Memory in Primary Care.

^a^Odds ratios report a 5-point difference in the scale score.
